# Prognostic Significance of the Bone Marrow-to-Aorta Uptake Ratio on 2-Deoxy-2-[^18^F]fluoro-d-glucose Positron Emission Tomography/Computed Tomography in Patients with Cholangiocarcinoma

**DOI:** 10.3390/biomedicines12050944

**Published:** 2024-04-24

**Authors:** Jeong Won Lee, Ik Dong Yoo, Sun-pyo Hong, Beodeul Kang, Jung Sun Kim, Yung Kil Kim, Sang Ho Bae, Su Jin Jang, Sang Mi Lee

**Affiliations:** 1 Department of Nuclear Medicine, College of Medicine, Soonchunhyang University Cheonan Hospital, 31 Suncheonhyang 6-gil, Dongnam-gu, Cheonan 31151, Republic of Korea; sads00@naver.com (J.W.L.);; 2 Division of Medical Oncology, Department of Internal Medicine, CHA Bundang Medical Center, CHA University, 59 Yatap-ro, Bundang-gu, Seongnam 13496, Republic of Korea; 3 Department of Surgery, College of Medicine, Soonchunhyang University Cheonan Hospital, 31 Suncheonhyang 6-gil, Dongnam-gu, Cheonan 31151, Republic of Korea; 4 Department of Nuclear Medicine, CHA Bundang Medical Center, CHA University, 59 Yatap-ro, Bundang-gu, Seongnam 13496, Republic of Korea

**Keywords:** bone marrow, cholangiocarcinoma, F-18 fluorodeoxyglucose, positron emission tomography, spleen

## Abstract

2-Deoxy-2-[^18^F]fluoro-d-glucose (FDG) uptake of the reticuloendothelial system on positron emission tomography/computed tomography (PET/CT) is known to be related to systemic inflammatory response to cancer cells in patients with diverse malignancies. This retrospective study aimed to investigate whether FDG uptake by the reticuloendothelial system had a prognostic value in predicting progression-free survival (PFS) and overall survival (OS) in 138 cholangiocarcinoma patients. Quantifying FDG uptake of the aorta, bone marrow (BM), liver, and spleen from staging FDG PET/CT images, we found significant correlations between the BM-to-aorta uptake ratio (BAR), spleen-to-aorta uptake ratio, and BM-to-liver uptake ratio with tumor stage and serum inflammatory markers. In the multivariate survival analysis, BAR was an independent predictor of PFS (*p* = 0.016; hazard ratio, 2.308) and OS (*p* = 0.030; hazard ratio, 2.645). Patients with stages III–IV of the disease and a high BAR exhibited low 1-year PFS (35.8%) and OS (60.2%) rates, while those with stages I–II of the disease and low BAR showed robust rates of 90.0% and 96.7%, respectively. BAR measured on staging FDG PET/CT might be a potential imaging biomarker offering insights into the systemic inflammatory response and predicting prognosis in cholangiocarcinoma. This study highlights BAR as a promising, independent predictor with potential for personalized prognostication and treatment strategies.

## 1. Introduction

Cholangiocarcinoma, originating in the biliary epithelium, represents the most prevalent malignant biliary tract disease [1,2]. Based on the anatomical location of the primary tumor lesion, it is categorized as intrahepatic cholangiocarcinoma (located proximal to the secondary branches of the left and right hepatic ducts), perihilar cholangiocarcinoma (located proximal to the origin of the cystic duct), or distal bile duct cancer (located in the common bile duct) [2]. Compared to Caucasian populations in Europe and the United States, the incidence of biliary tract cancer in Asian populations is quite high, ranking 8th place in the national cancer statistics of Korea [2,3]. Surgical resection is the sole curative treatment for cholangiocarcinoma, emphasizing cancer-free margins [4]. However, due to advanced-stage diagnoses, less than one-third of patients are deemed eligible for potentially curative tumor resections [4,5]. Furthermore, cholangiocarcinoma’s high chemoresistance and recurrence rates contribute to an unfavorable prognosis, with median survival rates of 8.0 months and 16.6 months for patients with intrahepatic and extrahepatic cholangiocarcinoma, respectively [4,6]. Therefore, non-invasive prognostic biomarkers that can stratify clinical outcomes and aid in determining treatment strategies are essential [7,8]. Cholangiocarcinomas are characterized by prominent desmoplastic stroma composed of various cell types, including immune cells [4]. Several recent studies have shown that immune cells play crucial roles in cholangiocarcinoma progression and treatment failure [4]. In clinical studies, increased serum inflammatory markers, such as the neutrophil-to-lymphocyte ratio (NLR) and platelet-to-lymphocyte ratio (PLR), which have been used to estimate systemic inflammatory response, were significantly associated with poor survival in patients with cholangiocarcinoma. This suggests that biomarkers of the immune response in the host could be used as prognostic factors [9,10].

In addition to conventional imaging modalities, 2-deoxy-2-[^18^F]fluoro-d-glucose (FDG) positron emission tomography/computed tomography (PET/CT) has been used for accurate staging, cancer recurrence detection, and predicting clinical outcomes in patients with cholangiocarcinoma [8,11,12]. Since FDG uptake by the organ also increases in an inflammatory condition, recent studies have revealed that the systemic inflammatory response to cancer cells can be estimated by FDG PET/CT images in patients with malignant diseases [13,14,15]. Previous studies showed a significant relationship between the degree of FDG uptake in the bone marrow (BM) and spleen, which are the main primary organs of the reticuloendothelial system, with immune cell infiltration in tumor tissue and serum inflammatory markers, and also revealed the significant prognostic value of FDG uptake in the BM and spleen for predicting survival in patients with diverse malignancies [14,15,16]. Given the significant role of immune cells in cholangiocarcinoma, FDG uptake by the reticuloendothelial system may also have prognostic significance in patients with cholangiocarcinoma. However, the existing literature reports only a single study indicating poor survival in patients with high splenic FDG uptake [17], and the clinical significance of FDG uptake in the BM remains unexplored in cholangiocarcinoma.

In this retrospective study, we investigated whether FDG uptake by the BM, liver, and spleen measured by staging with PET/CT holds significant prognostic value in predicting progression-free survival (PFS) and overall survival (OS) in patients with cholangiocarcinoma.

## 2. Materials and Methods

### 2.1. Patients

Patients who were histopathologically confirmed to have cholangiocarcinoma (intrahepatic cholangiocarcinoma, perihilar cholangiocarcinoma, and distal bile duct cancer) and underwent staging work-up examinations between January 2015 and May 2021 at three medical centers (CHA Bundang Medical Center, International St. Mary’s Hospital, and Soonchunhyang University Cheonan Hospital) were identified. Among these patients, the study included 138 patients with cholangiocarcinoma who underwent FDG PET/CT for staging and received either curative or palliative treatment. Exclusion criteria comprised patients with (1) a history of other malignant diseases or hematologic diseases, (2) concurrent infectious disease during staging work-up examinations, (3) malignant bile duct tumors other than cholangiocarcinoma, (4) those receiving supportive care without any treatment, and (5) individuals lost to follow-up within 12 months after treatment. In addition to FDG PET/CT, the initial staging work-up included blood tests and conventional imaging examinations, such as ultrasonography, contrast-enhanced abdominopelvic CT, and liver magnetic resonance imaging. The neutrophil-to-lymphocyte ratio (NLR) and platelet-to-lymphocyte ratio (PLR) were calculated from the blood cell count results by dividing the neutrophil count by the lymphocyte count and the platelet count by the lymphocyte count, respectively. Based on imaging examination results, tumor stages were assessed in accordance with the American Joint Committee on Cancer classification system, 8th edition.

### 2.2. Measurement of FDG PET/CT Parameters

FDG PET/CT scans were conducted using a Biograph mCT 20 scanner (Siemens Healthineers, Knoxville, TN, USA) at the International St Mary’s Hospital and a Biograph mCT 128 scanner (Siemens Healthineers) at the CHA Bundang Medical Center and Soonchunhyang University Cheonan Hospital. Patients were instructed to fast for at least 6 h before FDG injection. After confirmation of a peripheral blood glucose level below 200 mg/dL, FDG was intravenously injected 60 min prior to scanning as follows: 4.07 MBq/kg for a Biograph mCT 20 scanner (Siemens Healthineers) at the International St. Mary’s Hospital, 4.07 MBq/kg for a Biograph mCT 128 scanner (Siemens Healthineers) at the Soonchunhyang University Cheonan Hospital, and 5.18 MBq/kg for a Biograph mCT 128 scanner (Siemens Healthineers) at the CHA Bundang Medical Center. All PET/CT scans were conducted from the skull base to the proximal thigh with the patient in the supine position. A non-contrast-enhanced CT scan with a 5 mm slice thickness was performed using an automated dose modulation. Additionally, a PET scan lasting 1.5 min per bed position was conducted using a three-dimensional acquisition mode in all PET/CT scanners. PET images were reconstructed using an ordered subset expectation maximization algorithm with point spread function, time-of-flight modeling, and attenuation correction. 

**Figure 1:**
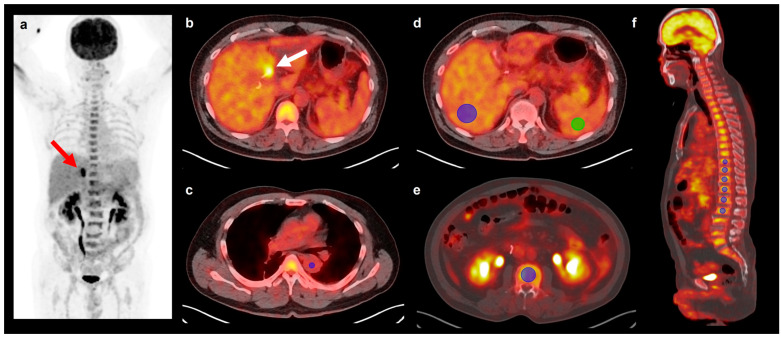
Maximum intensity projection image (**a**), transaxial images (**b**–**e**), and sagittal image (**f**) of FDG PET/CT showing VOIs for calculating FDG uptake of the aorta, bone marrow, liver, and spleen. A 60-year-old man underwent FDG PET/CT for staging work-up for histopathologically confirmed perhilar cholangiocarcinoma (arrows on (**a**,**b**)). A spherical 1 cm sized VOI was drawn in the intra-aorta area to measure FDG uptake of the aorta (blue circle in (**c**)). To measure FDG uptake of the liver and spleen, a spherical 3 cm sized VOI in the liver (blue circle in (**d**)) and a spherical 2 cm sized VOI in the spleen (green circle in (**d**)) were drawn. FDG uptake of bone marrow was measured by placing six spherical VOIs in the vertebral body of the thoracic and lumbar spines (blue circles in (**e**,**f**)). Because the patient had compression fractures in L3–4 spines, VOIs were drawn in T9–T12 spines and L1–2 spines.

FDG PET/CT images of all enrolled patients were retrospectively reviewed by consensus between at least two board-certified nuclear medicine physicians using the OsiriX MD 10.0 software (Pixmeo, Geneva, Switzerland). Reviewers could refer to findings from other conventional imaging studies but were blinded to additional clinical information and survival results. The maximum standardized uptake value (SUV) of the primary malignant tumor was measured by placing a manually drawn volume of interest (VOI) over the primary tumor lesion (tumor SUV). FDG uptake in the aorta, BM, liver, and spleen was measured using a previously published method [13,14,15] (Figure 1). The mean SUVs of the aorta, liver, and spleen were measured by drawing a spherical 1 cm VOI in the intra-aortic area (aorta SUV), a spherical 3 cm VOI in the liver while avoiding tumor lesions (liver SUV), and a spherical 2 cm VOI in the spleen (spleen SUV). Six spherical VOIs were drawn over the T10–T12 and L1–L3 spines, excluding spines that revealed pathological conditions, including compression fractures, post-operative changes, and severe osteoarthritic changes. Areas revealing an SUV higher than 75% of the maximum SUV for these six VOIs were automatically depicted. The mean SUV of the areas was calculated and defined as the mean SUV of BM (BM SUV). By using the values of the aorta SUV, the liver SUV, spleen SUV, and BM SUV, BM-to-aorta uptake ratio (BAR), spleen-to-aorta uptake ratio (SAR), liver-to-aorta uptake ratio (LAR), BM-to-liver uptake ratio (BLR), and spleen-to-liver uptake ratio (SLR) were measured.

### 2.3. Statistical Analysis

For continuous variables, the Shapiro–Wilk test assessed the normality of the distribution. The Kruskal–Wallis test examined differences in FDG PET/CT parameters of the reticuloendothelial system based on tumor classification. Post hoc analysis using Dunn’s test was conducted for variables exhibiting statistical significance in the Kruskal–Wallis test. Spearman’s correlation coefficient was calculated to investigate the relationship among the FDG PET/CT parameters of the reticuloendothelial system, TNM stage, and serum inflammatory markers. Univariate and multivariate survival analyses using Cox proportional hazards regression models were conducted to assess the prognostic significance of clinical factors and FDG PET/CT parameters of the reticuloendothelial system for predicting PFS and OS. Hazard ratios with Wald’s 95% confidence intervals (CIs) were calculated for variables in the univariate and multivariate survival analysis. Survival time was defined as the duration from initial treatment to the date of the detection of disease progression, death, or the last follow-up visit. Among the FDG PET/CT parameters of the reticuloendothelial system, variables showing statistical significance in the univariate analysis were included in the multivariate analysis, along with age, sex, tumor classification, TNM stage, and tumor SUV. Significantly correlated PET/CT parameters were included in separate multivariate models. Patients were classified into subgroups according to the combination of TNM stage and PET/CT parameters of the reticuloendothelial system. Additionally, PFS and OS were compared between the subgroups using the Cox proportional hazards regression model. To estimate survival curves, the optimal cut-off value was determined through receiver operating characteristic (ROC) curve analysis. The Kaplan–Meier method was employed for survival curve estimation, and survival curves between patient subgroups were compared using the log-rank test. In the comparison of survival curves among multiple patient subgroups, the Bonferroni correction was applied to account for multiple tests. All statistical analyses were performed using MedCalc Statistical Software version 22.016 (MedCalc Software Ltd., Ostend, Belgium). Statistical significance was set at *p* < 0.05.

## 3. Results

### 3.1. Characteristics of the Enrolled Patients

The clinical characteristics of 138 patients with cholangiocarcinoma are summarized in Table 1. Among the enrolled patients, 97 were male (70.3%), and 41 were female (29.7%), with a median age of 66 years. The primary tumors were classified as follows: 23 intrahepatic cholangiocarcinomas (16.7%), 60 perihilar cholangiocarcinomas (43.5%), and 55 distal bile duct cancers (39.9%). During staging work-up, 15 patients (10.9%) were diagnosed with stage IV cholangiocarcinoma. Surgical intervention was administered to 96 patients (69.6%), while the remaining 42 patients (30.4%) underwent treatment involving chemotherapy and/or radiotherapy. Among the surgically treated patients, 60 patients (62.5%) received adjuvant treatment after the surgery.

### 3.2. Correlation between PET/CT Parameters and Clinical Factors

The results of the correlation analysis of FDG PET/CT parameters of the reticuloendothelial system with tumor classification, tumor stage, and serum inflammatory markers are presented in Tables S1–S3. Correlation analysis with tumor classification revealed significant differences in the liver and spleen SUV between intrahepatic cholangiocarcinoma, perihilar cholangiocarcinoma, and distal bile duct cancer (*p* < 0.05), whereas none of the BM uptake parameters showed statistical significance (Table S1). Post hoc analysis revealed significantly higher SUVs of both the liver and spleen in perihilar cholangiocarcinoma compared to intrahepatic cholangiocarcinoma (*p* < 0.05; Figure S1). Correlation analysis with the TNM stage demonstrated significant correlations with BAR, SAR, and BLR (*p* < 0.05; Table S2). Patients with advanced tumor stages exhibited higher BAR, SAR, and BLR values (Figure S2).

In the correlation analysis with four serum inflammatory markers (C-reactive protein [CRP], white blood cell count, NLR, and PLR) (Table S3), both BAR and SAR demonstrated significant but weak positive correlations with the serum CRP level, NLR, and PLR (*p* < 0.05). Furthermore, BLR showed significant positive correlations with the serum CRP level, white blood cell count, and NLR (*p* < 0.05). In contrast to other PET/CT parameters that showed statistical significance in the correlation analysis, liver SUV was negatively correlated with the serum CRP level (*p* = 0.002; correlation coefficient = −0.314).

### 3.3. Survival Analysis 

The clinical follow-up duration ranged from 0.4 to 99.5 months, with the median RFS and OS of all patients at 14.8 months (95% CI, 10.6–19.6 months) and 38.9 months (95% CI, 26.4–39.4 months), respectively. Throughout the follow-up, 74 patients (53.6%) experienced cancer progression, and 58 patients (42.0%) died, indicating a 1-year RFS and 1-year OS of 54.6% (95% CI, 46.2–63.0%) and 74.8% (95% CI, 67.3–82.3%), respectively. The prognostic significance of FDG PET/CT parameters of the reticuloendothelial system and tumor SUV for predicting PFS and OS was assessed along with clinical factors. In univariate survival analysis (Table 2), increased BAR, SAR, and BLR were significantly associated with worse PFS and OS (*p* < 0.05). Tumor SUV was significantly associated only with OS (*p* = 0.046) and showed borderline statistical significance with PFS (*p* = 0.074). Of the clinical factors, T4 stage, lymph node metastasis, distant metastasis, and TNM stage were significant predictors of PFS and OS (*p* < 0.05). By contrast, none of the serum inflammatory markers were significant prognostic factors (*p* > 0.05). In the multivariate survival analysis for PFS and OS, age, sex, tumor classification, TNM stage, tumor SUV, and three PET/CT parameters of the reticuloendothelial system that showed statistical significance in the univariate analysis (BAR, SAR, and BLR) were incorporated (Table 3). Because BAR and BLR showed a significant correlation (*p* < 0.001; correlation coefficient = 0.765), both parameters were assessed in separate models. Multivariate analysis demonstrated that only BAR was an independent statistically significant predictor of PFS (*p* = 0.016; hazard ratio, 2.308; 95% CI, 1.169–4.557) and OS (*p* = 0.030; hazard ratio, 2.645; 95% CI, 1.101–6.354).

**Figure 2:**
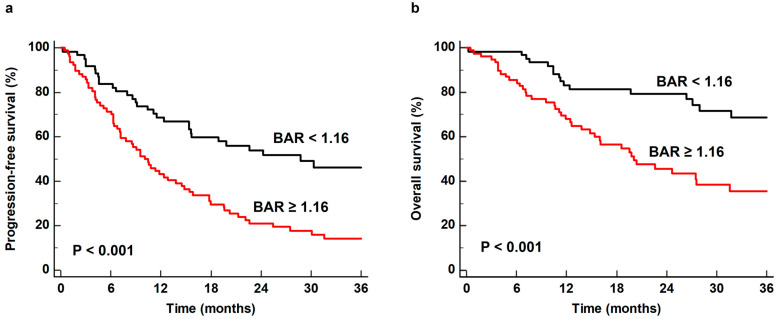
Kaplan–Meier curves of progression-free survival (**a**) and overall survival (**b**) according to the bone marrow-to-aorta uptake ratio (BAR).

For Kaplan–Meier analysis, BAR was categorized into two groups based on a cut-off value of 1.16, as determined by ROC curve analysis. Patients with BAR ≥ 1.16 showed significantly worse PFS and OS than those with BAR < 1.16 (*p* < 0.001 for both). The 1-year PFS and OS rates for patients with high BAR were 43.2% (95% CI, 32.0–54.4%) and 68.0% (95% CI, 57.1–78.9%), respectively, whereas patients with low BAR showed a 1-year PFS rate of 68.7% (95% CI, 57.0–80.4%) and 1-year OS rate of 83.1% (95% CI, 73.5–92.7%) (Figure 2).

**Figure 3:**
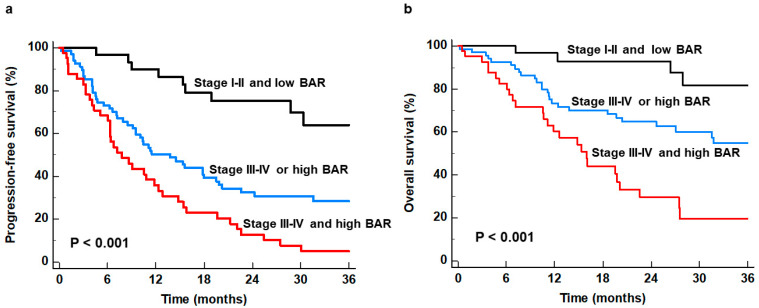
Kaplan–Meier curves of progression-free survival (**a**) and overall survival (**b**) according to TNM stage and bone marrow-to-aorta uptake ratio (BAR).

PFS and OS were further compared according to the TNM stage and BAR. Based on the combination of TNM stage and BAR, all patients were classified into three subgroups: (1) patients with stages I–II and BAR < 1.16, (2) patients with stages III–IV or BAR ≥ 1.16 (comprised to those with stages III–IV and BAR < 1.16 and those with stages I–II and BAR ≥ 1.16), and (3) patients with stages III–IV and BAR ≥ 1.16. In the comparative analysis of PFS and OS between the three patient subgroups, a *p*-value of <0.0167 (0.05/3) was considered statistically significant based on the Bonferroni correction. Of the three subgroups, patients with stages III–IV of the disease and a high BAR showed the worst PFS and OS (*p* < 0.001), displaying a 6.559-fold increased risk of disease progression and a 9.062-fold increased risk of death compared to patients with stages I-II of the disease and a low BAR (Table 4). The 1-year PFS and OS rates were 90.0% (95% CI, 80.2–99.8%) and 96.7% (95% CI, 90.3–100.0%) for patients with stages I–II of the disease and a low BAR, 50.3% (95% CI, 38.2–62.4%) and 73.4% (95% CI, 62.5–84.3%) for patients with stages III–IV of the disease or a high BAR, and 35.8% (95% CI, 20.9–50.7%) and 60.2% (95% CI, 44.4–76.0%) for patients with stages III–IV of the disease and a high BAR (Figure 3).

## 4. Discussion

In the present study, among the FDG PET/CT parameters of the reticuloendothelial system, BAR, BLR, and SAR correlated significantly with the TNM stage and serum inflammatory markers in patients with cholangiocarcinoma. In the univariate survival analysis, these three PET/CT parameters were also significantly associated with PFS and OS; however, in the multivariate survival analysis, only BAR was a significant independent prognostic factor for predicting PFS and OS. The combination of TNM stage and BAR can further stratify the risk of disease progression and death, demonstrating the worst survival in patients with stage III-IV cholangiocarcinoma and high BAR.

Over the decades, it has been continuously addressed that a significant proportion of patients with malignant diseases show diffusely increased FDG uptake in the BM and spleen, even higher than in the liver, irrespective of cancer type [14,17,18,19]. Since cancer-related inflammation has attracted the attention of cancer researchers in recent years, several studies have attempted to investigate whether FDG uptake in the reticuloendothelial system reflects the systemic inflammatory response to cancer cells in the host [13,14,20]. Three studies assessed the relationship of FDG uptake in the BM and spleen with the tumor immune microenvironment using the immunohistochemical analysis of surgical specimens [14,15,21]. In a study on uterine cervical cancer, significantly increased numbers of myeloid-derived suppressor cells in tumor tissues were observed in patients with high FDG uptake in the BM [15]. Another study on uterine cervical cancer showed that high splenic FDG uptake was related to high immune cell densities in the cancer tissue, especially for immune cells expressing CD20 (a marker of B lymphocytes) and CD68 (a marker of monocyte cell types, including macrophages) [21]. Furthermore, in a previous study on gastric cancer, FDG uptake in the BM and spleen revealed significant positive correlations with the degree of M2-type macrophages and CD8 T cell infiltration in the tumor tissue, respectively [14]. The results of these studies indicate that FDG uptake in the BM and spleen has a close and crucial relationship with the immune landscape in the tumor microenvironment [14,21]. Therefore, as also shown in the present study, it is hardly surprising that a significant correlation of FDG uptake in the BM and spleen with serum inflammatory markers such as CRP, NLR, and PLR has been consistently reported, suggesting the role of FDG uptake of the BM and spleen as an imaging biomarker for estimating the degree of systemic inflammatory response in patients with malignant diseases, including cholangiocarcinoma [19,22,23,24,25]. 

In a recent study, differences were observed in the tumor immune microenvironment among intrahepatic cholangiocarcinoma, perihilar cholangiocarcinoma, and distal bile duct cancer [26]. Multiplex immunohistochemical analysis showed that perihilar cholangiocarcinoma and distal bile duct cancer tended to exhibit a colder tumor immune microenvironment compared to intrahepatic cholangiocarcinoma [26]. In contrast, our study demonstrated a significantly higher value of the spleen SUV in patients with perhilar cholangiocarcinoma than in those with intrahepatic cholangiocarcinoma, with no significant differences noted in other PET/CT parameters of the BM and spleen. However, due to the limited number of patients with intrahepatic cholangiocarcinoma in this study, further research is required to investigate whether the PET/CT imaging parameters of the reticuloendothelial system vary based on the anatomical location of the cholangiocarcinoma. 

Notably, cholangiocarcinoma contains a large number of immunosuppressive immune cells in the tumor microenvironment [27]. Among these cells, M2-type macrophages, also known as tumor-associated macrophages, are the predominant immune cells in the tumor immune microenvironment of cholangiocarcinoma [4,27]. Tumor-associated macrophages inhibit inflammation and tissue repair while promoting tumor growth and metastasis [26,27]. Furthermore, the increased number of tumor-associated macrophages in the tumor tissue correlated with increased risk of recurrence and reduced OS in cholangiocarcinoma [26,27,28]. CD8 T cells are well-known immune cells that play a crucial role in anti-cancer immune response, exhibiting an increased density in the tumor tissue in cholangiocarcinoma patients with a favorable prognosis [27,29]. However, in a recent study, a lower density of CD8 T cells expressing the programmed death ligand 1 was significantly associated with improved survival in patients with cholangiocarcinoma [26]. Considering the significant relationship between FDG uptake in the BM and spleen and immune cell infiltration in tumor tissues, especially macrophages [14,21], we speculate that FDG uptake in the BM and spleen could have prognostic significance in predicting survival in patients with cholangiocarcinoma. In our study, the results of univariate survival analysis showed that BAR, BLR, and SAR were significantly associated with both PFS and OS and, for multivariate analysis, only BAR remained an independent prognostic factor for predicting PFS and OS, indicating worse survival in patients with a high BAR. These results suggest that FDG uptake in the BM, rather than the spleen, might be a more suitable imaging biomarker for estimating the degree of systemic inflammatory response and predicting prognosis in patients with cholangiocarcinoma. Due to the abundance of immunosuppressive immune cells in the tumor microenvironment, the efficacy of immunotherapy has been limited in patients with cholangiocarcinoma [27]. However, numerous numbers of preclinical and clinical studies have explored diverse immunotherapies with novel treatment targets [27]. In future studies, BAR might aid in identifying candidates for immunotherapy and predicting treatment outcomes in patients with cholangiocarcinoma. 

In addition to BM SUV, BLR, and BAR have been used as PET/CT imaging parameters for the BM to mitigate the variations in BM FDG uptake among individuals [13,15,30]. Several previous studies have demonstrated that BLR exhibited superior prognostic value compared to BM SUV in predicting clinical outcomes across various types of cancers, including head and neck cancer, breast cancer, non-small cell lung cancer, and gastric cancer, and only a single study focusing on cervical cancer demonstrated the significant prognostic value of BAR [14,15,19,31]. However, the results of this study indicate the superior prognostic value of BAR compared to that of BLR. This discrepancy might stem from cholangiocarcinoma’s potential to induce biliary obstruction and cholestatic liver injury, affecting FDG uptake of the liver [32,33]. Furthermore, our study revealed that FDG uptake in the normal liver tissue was influenced by cholangiocarcinoma tumor classification, suggesting that BAR might be a more suitable imaging parameter than BLR for estimating FDG uptake in the BM in patients with cholangiocarcinoma. 

Although BAR was an independent predictor of PFS and OS in our study, it only represented the degree of inflammatory response of the host and did not reflect the tumor burden. To overcome this limitation, previous studies combined the imaging parameters of cancer lesions and BM, demonstrating improved prognostic prediction compared to tumor factors or FDG uptake of BM alone [20,34]. Our study further demonstrated that combining the TNM stage and BAR allows for the stratification of disease progression and death. The patient subgroup with stages I–II of the disease and low BAR exhibited 1-year PFS and OS rates ≥ 90%, whereas those with stages III–IV of the disease with a high BAR had markedly lower rates at 35.8% and 60.2%, respectively. Therefore, cholangiocarcinoma patients with advanced tumor stage and increased systemic inflammatory response necessitate intense treatment with close surveillance. 

The current study had several limitations. Firstly, because the study was retrospectively performed, there might have been a selection bias that could have influenced the results. Secondly, we did not include all cholangiocarcinoma types (intrahepatic cholangiocarcinoma, perihilar cholangiocarcinoma, and distal ductal cancer) in our analysis. Since different immune landscapes have been shown between subtypes of cholangiocarcinoma in previous studies [26,29], the prognostic impact of FDG uptake by the reticuloendothelial system could be different between subtypes. Third, though PET/CT scanners used in this study were made by the same company, the use of two different dosages of FDG for the enrolled patients might affect the results. Lastly, the relationship between FDG PET/CT parameters of the reticuloendothelial system and the tumor immune microenvironment was studied primarily in gastric and cervical cancers [14,15,21]. Given the unique tumor immune microenvironment of cholangiocarcinoma, further studies using flow cytometry and animal models are imperative to elucidate its underlying mechanism.

## 5. Conclusions

In patients diagnosed with cholangiocarcinoma, BAR serves as an independent prognostic factor for predicting PFS and OS. BAR exhibited a significant and positive correlation with serum inflammatory markers. Patients characterized as having a high BAR demonstrated inferior survival rates compared to those with a low BAR. The combination of TNM stage and BAR could further enhance prognostic value, revealing that patients with stages III–IV of the disease and a high BAR experience the most unfavorable survival outcomes. The utilization of BAR on FDG PET/CT might be a potential imaging biomarker to assess the degree of systemic inflammatory response and predict prognosis in patients with cholangiocarcinoma.

## Figures and Tables

**Table 1:** Patient characteristics.

Variables		Number of Patients (%)
Age (years)		66 (35–89) *
Sex	Men	97 (70.3%)
	Women	41 (29.7%)
Tumor classification	Intrahepatic	23 (16.7%)
	Perihilar	60 (43.5%)
	Distal bile duct	55 (39.9%)
T stage	T1	22 (15.9%)
	T2	43 (31.2%)
	T3	38 (27.5%)
	T4	35 (25.4%)
Lymph node metastasis	Negative	81 (58.7%)
	Positive	57 (41.3%)
Distant metastasis	Negative	120 (87.0%)
	Positive	18 (13.0%)
TNM stage	Stage I	14 (10.1%)
	Stage II	52 (37.7%)
	Stage III	57 (41.3%)
	Stage IV	15 (10.9%)
Blood tests	CA19-9 (U/mL)	151.2 (0.8–14,456.0) *
	CRP (mg/dL)	4.15 (0.03–1030.0) *
	WBC (×10^12^ cells/L)	6.97 (3.38–24.68) *
	NLR	3.14 (0.99–3.98) *
	PLR	176.31 (38.54–670.47) *
FDG PET/CT parameters	Tumor SUV	5.97 (2.72–17.93) *
	BM SUV	2.14 (1.10–3.99) *
	Liver SUV	2.49 (1.51–3.69) *
	Spleen SUV	2.07 (1.17–5.29) *
	BAR	1.20 (0.68–2.33) *
	LAR	1.39 (1.04–2.34) *
	SAR	1.18 (0.82–2.09) *
	BLR	0.85 (0.48–1.91) *
	SLR	0.85 (0.58–1.66) *

* Median (range). BAR, bone marrow-to-aorta uptake ratio; BLR, bone marrow-to-liver uptake ratio; BM, bone marrow; CA19-9, carbohydrate antigen 19-9; CRP, C-reactive protein; LAR, liver-to-aorta uptake ratio; NLR, neutrophil-to-lymphocyte ratio; PLR, platelet-to-lymphocyte ratio; SAR, spleen-to-aorta uptake ratio; SLR, spleen-to-liver uptake ratio; SUV, standardized uptake ratio; WBC, white blood cell.

**Table 2:** Prognostic significance of clinical factors and PET/CT parameters for progression-free survival (PFS) and overall survival (OS) in univariate survival analysis.

Variables		PFS		OS	
		*p*-Value	Hazard Ratio (95%CI)	*p*-Value	Hazard Ratio (95% CI)
Age (1-year increase)		0.166	0.986 (0.966–1.006)	0.204	0.983 (0.958–1.009)
Sex (women vs. men)		0.847	0.960 (0.638–1.446)	0.839	1.055 (0.627–1.775)
Tumor classification (intrahepatic vs.)	Perihilar	0.657	0.881 (0.502–1.545)	0.939	1.028 (0.501–2.106)
	Distal bile duct	0.092	0.647 (0.362–1.157)	0.176	0.586 (0.270–2.106)
T stage (T1 vs.)	T2	0.038	2.204 (1.043–4.658)	0.257	1.708 (0.677–4.304)
	T3	0.125	1.818 (0.848–3.895)	0.515	1.375 (0.528–3.581)
	T4	<0.001	3.639 (1.725–7.677)	0.019	2.980 (1.195–7.433)
Lymph node metastasis (negative vs. positive)		<0.001	2.144 (1.424–3.226)	<0.001	2.500 (1.481–4.219)
Distant metastasis (negative vs. positive)		<0.001	2.632 (1.563–4.430)	<0.001	3.351 (1.784–6.295)
TNM stage (stage I–II vs.)	Stage III	0.003	1.996 (1.262–3.063)	0.019	2.011 (1.122–3.604)
	Stage IV	<0.001	3.624 (1.967–6.678)	<0.001	4.616 (2.172–9.810)
CA19-9 (1.0 U/mL increase)		0.139	1.000 (1.000–1.001)	0.373	1.000 (1.000–1.001)
CRP (1.0 mg/dL increase)		0.773	1.000 (0.999–1.002)	0.645	0.999 (0.996–1.002)
WBC (1.0 × 10^12^ cells/L increase)		0.377	1.027 (0.968–1.091)	0.071	1.067 (0.995–1.144)
NLR (1.0 increase)		0.658	1.002 (0.942–1.065)	0.905	1.005 (0.926–1.091)
PLR (1.0 increase)		0.936	0.999 (0.998–1.002)	0.659	0.999 (0.997–1.002)
Tumor SUV (1.0 increase)		0.074	1.048 (0.995–1.104)	0.046	1.067 (1.001–1.136)
BM SUV (1.0 increase)		0.065	1.395 (0.979–1.988)	0.305	1.286 (0.795–2.080)
Liver SUV (1.0 increase)		0.834	0.955 (0.619–1.473)	0.296	0.745 (0.429–1.295)
Spleen SUV (1.0 increase)		0.649	1.011 (0.726–1.407)	0.360	0.790 (0.477–1.309)
BAR (1.0 increase)		<0.001	2.850 (1.539–5.276)	0.003	3.370 (1.520–7.470)
LAR (1.0 increase)		0.066	3.132 (0.926–10.596)	0.112	3.106 (0.769–12.538)
SAR (1.0 increase)		0.044	2.135 (1.018–4.478)	0.048	2.635 (1.001–6.947)
BLR (1.0 increase)		0.013	2.806 (1.242–6.342)	0.033	3.214 (1.098–9.408)
SLR (1.0 increase)		0.664	1.277 (0.424–3.847)	0.947	0.950 (0.206–4.378)

BAR, bone marrow-to-aorta uptake ratio; BLR, bone marrow-to-liver uptake ratio; BM, bone marrow; CA19-9, carbohydrate antigen 19-9; CI, confidence interval; CRP, C-reactive protein; LAR, liver-to-aorta uptake ratio; NLR, neutrophil-to-lymphocyte ratio; PLR, platelet-to-lymphocyte ratio; SAR, spleen-to-aorta uptake ratio; SLR, spleen-to-liver uptake ratio; SUV, standardized uptake ratio; WBC, white blood cell.

**Table 3:** Prognostic significance of PET/CT parameters for progression-free survival (PFS) and overall survival (OS) in multivariate survival analysis.

Variables		PFS				OS			
		Model 1		Model 2		Model 1		Model 2	
		*p*-Value	Hazard Ratio (95% CI)	*p*-Value	Hazard Ratio (95% CI)	*p*-Value	Hazard Ratio (95% CI)	*p*-Value	Hazard Ratio (95% CI)
Age (1-year increase)		0.669		0.882		0.609		0.753	
Sex (women vs. men)		0.540		0.561		0.340		0.356	
Tumor classification (intrahepatic vs.)	Perihilar	0.470		0.449		0.308		0.271	
	Distal bile duct	0.224		0.194		0.466		0.410	
TNM stage (stage I–II vs.)	Stage III	0.004	1.938 (1.243–3.019)	0.004	2.451 (1.325–4.535)	0.019	2.019 (1.125–3.622)	0.026	1.969 (1.085–3.576)
	Stage IV	0.002	2.828 (1.490–5.367)	0.001	4.436 (1.781–11.049)	0.001	3.592 (1.639–7.875)	<0.001	3.926 (1.805–8.542)
Tumor SUV (1.0 increase)		0.324		0.293		0.173		0.148	
BAR (1.0 increase)		0.016	2.308 (1.169–4.557)	-	-	0.030	2.645 (1.101–6.354)	-	-
SAR (1.0 increase)		0.337		0.167		0.270		0.137	
BLR (1.0 increase)		-	-	0.221		-	-	0.165	

BAR, bone marrow-to-aorta uptake ratio; BLR, bone marrow-to-liver uptake ratio; CI, confidence interval; SAR, spleen-to-aorta uptake ratio; SUV, standardized uptake ratio.

**Table 4:** Comparison of progression-free survival (PFS) and overall survival (OS) between patient subgroups classified by TNM stage and BAR.

Subgroups	PFS		OS	
	*p*-Value	Hazard Ratio (95% CI)	*p*-Value	Hazard Ratio (95% CI)
Stage I–II and low BAR	-	1.00	-	1.00
Stage III–IV or high BAR	0.004	3.662 (1.794–7.475)	0.012	3.814 (1.337–10.877)
Stage III–IV and high BAR	<0.001	6.559 (3.148–13.667)	<0.001	9.062 (3.138–26.171)

BAR, bone marrow-to-aorta uptake ratio; CI, confidence interval.

## Data Availability

The datasets generated during and/or analyzed during the current study are available from the corresponding authors upon reasonable request.

## Supplementary Materials

The following supporting information can be downloaded at https://www.mdpi.com/article/10.3390/biomedicines12050944/s1, Table S1: Correlation analysis of FDG PET/CT parameters of reticuloendothelial system with tumor classification; Table S2: Correlation analysis of FDG PET/CT parameters of reticuloendothelial system with TNM stage; Table S3: Correlation analysis of FDG PET/CT parameters of reticuloendothelial system with serum inflammatory makers; Figure S1: Distribution of liver SUV (a) and spleen SUV (b) in patients with intrahepatic cholangiocarcinoma, perihilar cholangiocarcinoma, and distal bile duct cancer; Figure S2: Distribution of BAR (a), SAR (b), and BLR (c) according to TNM stage.
