# Supplementary material for: Prognostic Significance of the Bone Marrow-to-Aorta Uptake Ratio on 2-Deoxy-2-[18F]fluoro-d-glucose Positron Emission Tomography/Computed Tomography in Patients with Cholangiocarcinoma
Source: Biomedicines. 2024 Apr 24;12(5):944. doi: 10.3390/biomedicines12050944 (PMC11118843; doi:10.3390/biomedicines12050944)

Table S1. Correlation analysis of FDG PET/CT parameters of reticuloendothelial system with tumor classification.

Variables	Intrahepatic cholangiocarcinoma (n=23)	Perihilar cholangiocarcinoma (n=60)	Distal bile duct cancer (n=55)	P-value
BM SUV	2.12 (1.78–2.36)	2.00 (1.77–2.54)	2.21 (1.85–2.52)	0.672
**Liver SUV**	**2.32 (2.15–2.63)**	**2.63 (2.20–3.01)**	2.48 (2.22–2.83)	**0.034***
**Spleen SUV**	**1.91 (1.77–2.04)**	**2.25 (1.81–2.57)**	2.18 (1.88–2.42)	**0.026***
BAR	1.27 (1.09–1.52)	1.16 (1.00–1.37)	1.21 (1.09–1.31)	0.352
LAR	1.39 (1.27–1.48)	1.42 (1.30–1.52)	1.36 (1.29–1.46)	0.467
SAR	1.18 (1.03–1.28)	1.23 (1.06–1.38)	1.16 (1.06–1.29)	0.358
BLR	0.91 (0.80–1.03)	0.82 (0.71–0.95)	0.88 (0.78–0.97)	0.127
SLR	0.83 (0.76–0.91)	0.86 (0.77–0.92)	0.85 (0.78–0.94)	0.709

Expressed in median (interquartile range)

*On post-hoc analysis, there were significant differences of liver SUV and spleen SUV between patients with intrahepatic and perihilar cholangiocarcinoma (p<0.05).

BAR, bone marrow-to-aorta uptake ratio; BLR, bone marrow-to-liver uptake ratio; BM, bone marrow; LAR, liver-to-aorta uptake ratio; SAR, spleen-to-aorta uptake ratio; SLR, spleen-to-liver uptake ratio; SUV, standardized uptake ratio

Table S2. Correlation analysis of FDG PET/CT parameters of reticuloendothelial system with TNM stage.

Variables	P-value	Correlation Coefficient	Stage I	Stage II	Stage III	Stage IV
BM SUV	0.167	0.195	2.02 (1.53–2.48)	2.13 (1.78–2.44)	2.14 (1.88–2.59)	2.15 (1.79–2.62)
Liver SUV	0.446	-0.065	2.57 (2.33–3.09)	2.46 (2.19–2.78)	2.70 (2.16–2.99)	2.24 (1.88–2.37)
Spleen SUV	0.684	0.135	2.32 (1.90–2.53)	2.07 (1.77–2.32)	2.13 (1.92–2.58)	1.86 (1.65–2.14)
**BAR**	**0.002**	**0.261**	**0.99 (0.92–1.23)**	**1.21 (1.08–1.30)**	**1.15 (1.05–1.40)**	**1.47 (1.26–1.61)**
LAR	0.065	0.157	1.33 (1.30–1.41)	1.37 (1.27–1.47)	1.44 (1.30–1.53)	1.41 (1.29–1.48)
**SAR**	**0.011**	**0.216**	**1.14 (1.03–1.16)**	**1.14 (1.05–1.32)**	**1.27 (1.08–1.42)**	**1.22 (1.16–1.30)**
**BLR**	**0.015**	**0.206**	**0.71 (0.67–0.95)**	**0.86 (0.78–0.96)**	**0.84 (0.75–1.00)**	**1.02 (0.88–1.12)**
SLR	0.291	0.091	0.83 (0.79–0.87)	0.83 (0.76–0.92)	0.87 (0.76–0.96)	0.84 (0.81–0.91)

Expressed in median (interquartile range)

BAR, bone marrow-to-aorta uptake ratio; BLR, bone marrow-to-liver uptake ratio; BM, bone marrow; LAR, liver-to-aorta uptake ratio; SAR, spleen-to-aorta uptake ratio; SLR, spleen-to-liver uptake ratio; SUV, standardized uptake ratio

Table S3. Correlation analysis of FDG PET/CT parameters of reticuloendothelial system with serum inflammatory makers.

Variables		CRP	WBC	NLR	PLR
BM SUV	P-value	0.978	0.230	**0.017**	0.597
	Correlation coefficient	0.002	0.103	**0.203**	0.045
Liver SUV	P-value	**0.002**	0.324	0.885	0.629
	Correlation coefficient	**-0.314**	-0.085	0.012	-0.041
Spleen SUV	P-value	0.344	0.536	0.156	0.507
	Correlation coefficient	-0.081	-0.053	0.121	0.057
BAR	P-value	**<0.001**	**0.036**	**0.005**	**0.035**
	Correlation coefficient	**0.292**	**0.178**	**0.240**	**0.180**
LAR	P-value	0.646	0.914	0.270	0.095
	Correlation coefficient	0.039	0.009	0.094	0.143
SAR	P-value	**0.002**	0.771	**0.045**	**0.007**
	Correlation coefficient	**0.264**	-0.025	**0.164**	**0.228**
BLR	P-value	**<0.001**	**0.037**	**0.007**	0.187
	Correlation coefficient	**0.283**	**0.178**	**0.230**	0.113
SLR	P-value	**0.002**	0.960	0.123	0.053
	Correlation coefficient	**0.266**	-0.004	0.132	0.165

BAR, bone marrow-to-aorta uptake ratio; BLR, bone marrow-to-liver uptake ratio; BM, bone marrow; CRP, C-reactive protein; LAR, liver-to-aorta uptake ratio; NLR, neutrophil-to-lymphocyte ratio; PLR, platelet-to-lymphocyte ratio; SAR, spleen-to-aorta uptake ratio; SLR, spleen-to-liver uptake ratio; SUV, standardized uptake ratio; WBC, white blood cell

Figure S1. Distribution of liver SUV (a) and spleen SUV (b) in patients with intrahepatic cholangiocarcinoma, perihilar cholangiocarcinoma, and distal bile duct cancer.

(SUV, standardized uptake value)

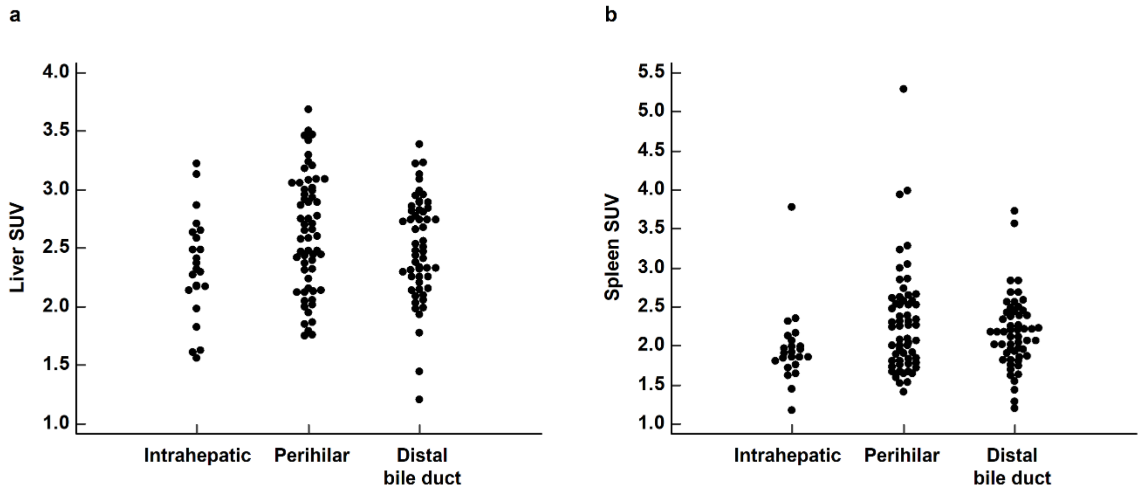

Figure S2. Distribution of BAR (a), SAR (b), and BLR (c) according to TNM stage.

(BAR, bone marrow-to-aorta uptake ratio; BLR, bone marrow-to-liver uptake ratio; SAR, spleen-to-aorta uptake ratio)

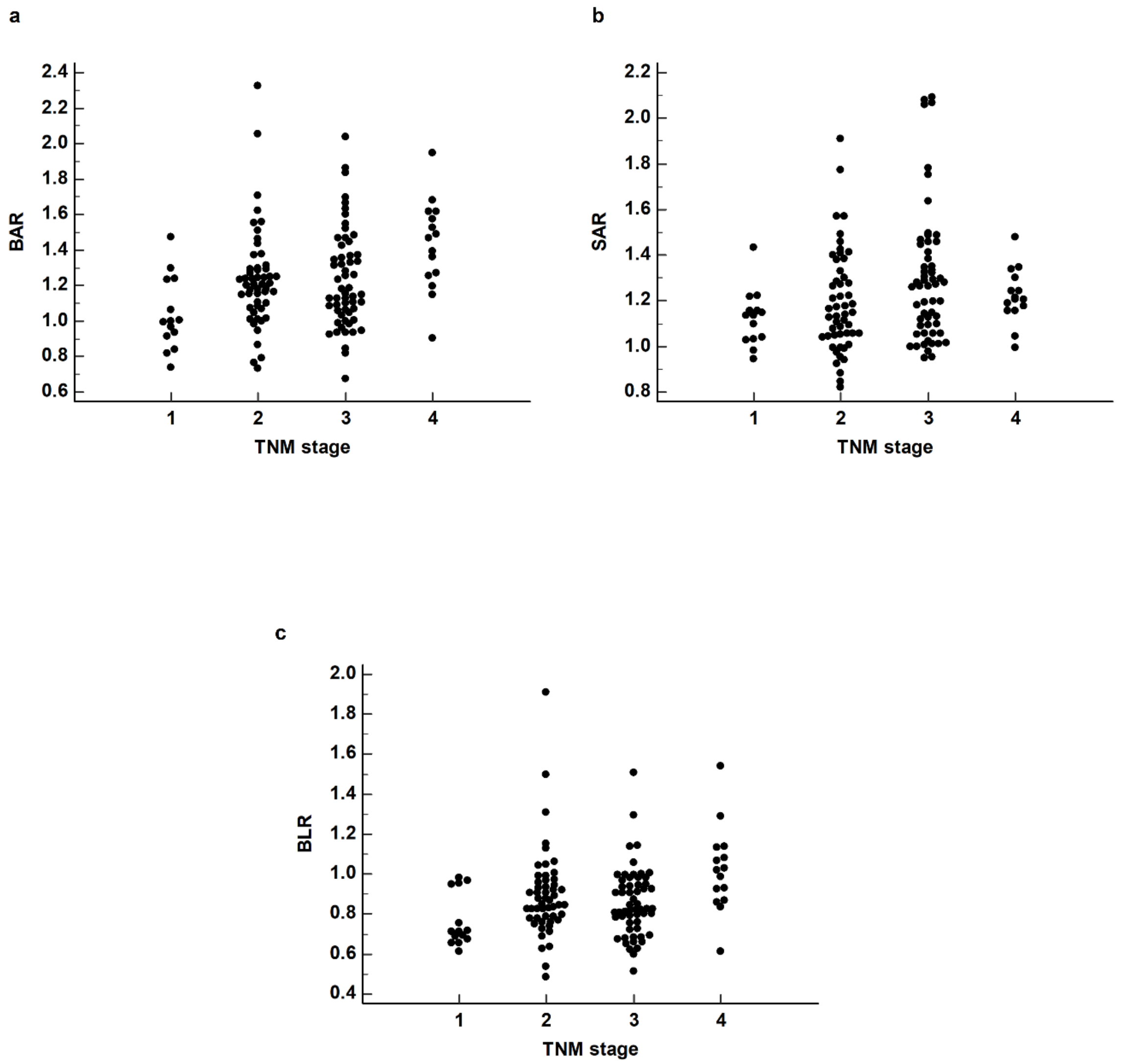

Supplement: Supplementary file 1 [file biomedicines-12-00944-s001.zip › biomedicines-2913189-supplementary.pdf]
